# Predicting Depression in Parkinson’s Disease Using Commonly Available PD Questionnaires

**DOI:** 10.3390/jcm13072069

**Published:** 2024-04-03

**Authors:** Emanuele Camerucci, Kelly E. Lyons, Rajesh Pahwa

**Affiliations:** Department of Neurology, University of Kansas Medical Center, Kansas City, KS 66160, USA; klyons@kumc.edu (K.E.L.);

**Keywords:** Parkinson’s disease, depression, UPDRS

## Abstract

**Background**: Depression is common in patients with Parkinson’s disease (PD) and significantly impacts both the patients and their caregivers. The associations between depression and the responses from commonly used questionnaires for PD patients were assessed. New patients presenting to the Movement Disorder Center completed a number of questionnaires, including assessments of the motor and non-motor symptoms of PD, including depression. **Methods**: The PD patients were grouped according to severity of depression: none, mild, and moderate–severe, based on the Geriatric Depression Scale (GDS) scores. The mean scores of the Unified PD Rating Scale (UPDRS), Montreal Cognitive Assessment (MoCA), Epworth Sleepiness Scale (ESS), Non-motor Symptoms Scale (NMSS), PD Quality of life (PDQ-39), Hoehn and Yahr score (H&Y), levodopa equivalent daily dose (LEDD), and number of antidepressants used were collected. There were 1214 PD patients included. **Results:** Increasing depression scores were associated with worsening motor symptoms (according to the UPDRS and H&Y), non-motor symptoms (according to the NMSS), cognition (according to the MoCA), sleepiness (according to the ESS), and quality of life (according to the PDQ-39) (all *p*-values of *p* < 0.001). Only half of the patients with mild or moderate–severe depression were taking antidepressants, and the LEDD increased with depression severity. The risk of depression increased by 16% and 5% for every 1-point increase in the NMSS and PDQ-39 scores, respectively. **Conclusions:** Depression is often unrecognized and undertreated and should be assessed regularly in PD patients, especially in those who demonstrate changes in motor or non-motor symptoms.

## 1. Introduction

Parkinson’s disease (PD) is a neurodegenerative disorder marked by four cardinal motor manifestations: resting tremor, rigidity, bradykinesia, and postural instability. Pathologically, it is characterized by the accumulation of alpha-synuclein aggregates in various structures in the central and autonomous nervous system. This condition, mostly known for its motor symptoms, embodies a multifaceted syndrome also characterized by an array of non-motor symptoms (NMSs). It is estimated that 100% of PD patients have at least one NMS [1], which encompasses neuropsychiatric symptoms (e.g., anxiety and depression), sleep disorders (e.g., REM sleep behavior disorder, insomnia, excessive daytime sleepiness, and obstructive sleep apnea), gastrointestinal dysfunction (e.g., constipation), sensory disturbances (e.g., anosmia), and executive and autonomic dysfunction (e.g., orthostatic hypotension) [1]. The NMSs exert a significant impact on the quality of life not only of the patients but also of caregivers and their families. One of the biggest hurdles for PD patients is NMSs that do not respond to antiparkinsonian medications [2]. For instance, depressive symptoms can manifest in up to 80% of individuals affected by PD and exert a negative effect on the quality of life of both patients and caregivers [3]. Depression remains amenable to potential therapeutic interventions that aim at increasing the neurotransmitter levels within the central nervous system, using compounds such as reuptake inhibitors of serotonin and/or norepinephrine, as well as dopamine agonists. Among these medications, evidence suggests that reuptake inhibitors of serotonin should be considered as first-line agents. The literature has shown potential benefits when patients used reuptake inhibitors of serotonin such as paroxetine and citalopram, in addition to other agents (nortriptyline and venlafaxine) [4].

Non-pharmacological interventions may be beneficial, such as physical activities, the arts [5,6], and commonly available substances such as essential oils [7]. Although depression is a common feature of PD and of the general population, diagnosing it is not as immediate as it may seem. The complexity of diagnosing depression arises from the resemblance of certain traits of depression—such as psychomotor slowness, hypomimia, fatigue, apathy, and sleep disturbances—to motor symptoms of PD, thus leading to its under-recognition and possibly to inadequate treatment. Therefore, it is imperative to conscientiously screen for depressive symptoms on an annual basis, ensuring timely intervention as warranted.

When depression is not recognized and treated, patients may experience symptoms that encompass multiple domains, including functional disability, cognitive impairment, and mortality, together with a worse quality of life in both patient and caregiver [8].

In this study, using a large cohort of individuals at their first visit to a tertiary specialized center, the goal was to assess if commonly used screening questionnaires for PD were able to predict the presence of depression, in order to determine the prevalence of depression and to determine how depression relates to other non-motor and motor symptoms of PD.

## 2. Methods

### 2.1. Study Design and Population

This is a descriptive study examining the relationship between the various assessments collected as part of the routine clinical care of PD patients at their initial clinic visit. New patients, commonly referred by community-based neurologists or general practitioners, undergo a comprehensive assessment at their first visit to the movement disorder clinic. This entails the completion of diverse questionnaires addressing motor and non-motor aspects of PD, coupled with the compilation of their medical history, concurrent medical conditions, therapeutic regimens, and dosages. All patients signed written informed consent for their clinical information to be databased and used for research. This study was conducted in accordance with the Declaration of Helsinki, and approved by the Institutional Review Board of the University of Kansas Medical Center (protocol code 12351, approved on 2 June 2023). All patients with PD, which was confirmed after evaluation by a movement disorder specialist (RP) and a complete Geriatric Depression Scale [9], were included in the study. Patients with neurological conditions in addition to PD and those with incomplete data were not included.

### 2.2. Measures

1. Geriatric Depression Scale (GDS [9]): There are 30 patient-completed questions in this assessment, which was first developed in the early 1980s to assess depressive symptoms in the general geriatric population. Scores range from a minimum of 0 to a maximum of 30. The PD patients were divided into three groups based on their score on the GDS questionnaire. The patients were stratified based on their depression level, according to what has been previously reported [10]. The three groups were the following: no depression (GDS, 0–9); mild depression (GDS, 10–19); and moderate–severe depression (GDS, 20–30). The GDS was used in this population of largely older adults as the GDS has been shown to be simpler for older adults while efficiently screening for depression [11].
2. Unified Parkinson’s Disease Rating Scale (UPDRS [12]): This is a questionnaire divided into four sections, in which the higher scores represent greater PD-related disability/severity: mentation (four historical questions regarding cognition, hallucinations, depression, and apathy, with responses ranging from 0–4 for a maximum score of 12); activities of daily living (ADLs) (13 historical questions associated with daily activities, with responses ranging from 0–4 for a maximum score of 52); motor (14 assessments of motor functioning completed by the investigator, with responses for each item ranging from 0–4 with a maximum score of 108); and complications (questions about motor complications, nausea/vomiting, sleep, and orthostatic hypotension). The total score is the sum of the mentation, ADLs, and motor sections.
3. Non-motor Symptoms Scale (NMSS [13]): This is a patient-completed scale with 30 yes/no questions focusing on the presence or absence of various NMSs.
4. Montreal Cognitive Assessment (MoCA [14]): This is a brief cognitive screening that examines visuospatial/executive functioning, naming, attention, language, abstraction, delayed recall, and orientation. The lower scores represent greater cognitive impairment, and the total possible score is 30. The cut-off for cognitive impairment is a score of 24.
5. Epworth Sleepiness Scale (ESS [15]): This is a patient-completed eight-question assessment of daytime sleepiness. Each question ranges from 0–3, with the higher scores representing greater daytime sleepiness. The total possible score is 24.
6. Parkinson’s Disease Questionnaire (PDQ-39 [16]): This is a patient-completed, 39-item questionnaire used to assess the quality of life in PD patients. Each question ranges from 0–4, with the lower scores representing a better quality of life. The PDQ assesses eight domains including mobility, ADLs, emotional well-being, stigma, social support, cognition, communication, and bodily discomfort. The total score and subscores are transformed into percentages ranging from 0–100.
7. Hoehn and Yahr scale (H&Y [17]): This is a five-point scale for clinically staging PD, where 0 is no disability, 1 is a unilateral disease, 2 is a bilateral disease, 3 is a bilateral disease with impairment in balance, 4 is disabling disease but able to walk/stand unassisted, and 5 is bed- or wheelchair-bound.
8. Levodopa equivalent daily dose (LEDD) [18]: This is a conversion score for computing the amount of various dopaminergic medications to a daily equivalence of levodopa (in mg).
9. The number of antidepressants used was counted based on the class of action. Any different kind of medication used was counted as one, regardless of the dosage and/or when it was started. The medications were divided as follows: SSRIs (escitalopram, citalopram, sertraline, paroxetine, and fluoxetine), SNRIs (venlafaxine and duloxetine), and “other antidepressants” (nefazodone, trazodone, amytriptiline, nortriptiline, mirtazapine, and buproprion). Under the category of “any antidepressants”, all the above medications were included.

### 2.3. Statistical Analysis

All categorical variables were reported as frequency counts and percentages, and all continuous variables were reported as means and ranges. The PD patients who did not have GDS questionnaire scores available were excluded from the analysis. 

The mean scores and ranges on all remaining questionnaires (UPDRS, NMSS, MoCA, ESS, PDQ-39, H&Y, and LEDD) were obtained and broken down by GDS group. A Kruskal–Wallis test was used to test for the differences of any given questionnaire between the three GDS groups. A post hoc pairwise analysis was conducted with the Wilcoxon Rank Sum Test. The differences in the usage of medications (broken down by category) between the three GDS groups were compared with the Chi-Square Test.

A logistic regression model was created to assess the risk of developing depression by changing the other questionnaires’ scores. In order to run such a model, a dichotomous definition of depression vs. no depression based on GDS score was needed. However, this has not yet been established in the literature. Therefore, the cut-off has arbitrarily ranged between 7 and 14. A cut-off of 14 could represent the cut-off to minimize false positives, maxing out specificity at the cost of sensitivity [10]. Hence, for this study, we used the most conservative approach and used a cut-off of 14 for defining depression. All statistical analyses were performed with GraphPad Prism 8.0.0 (GraphPad Software, San Diego, CA, USA). The level of significance was set at *p* < 0.05.

## 3. Results

### 3.1. Questionnaire Scores Based on GDS Groups

A total of 1214 patients were confirmed as PD with 90–100% confidence by a movement disorder specialist (RP) and were included. There were 455 women and 759 men. The mean (range) age at PD onset was 63 (55–69) years, and the mean age at PD diagnosis was 64 (57–70) years. Detailed information regarding demographic variables, broken down by GDS group, is included in Table 1.

**Figure 1:**
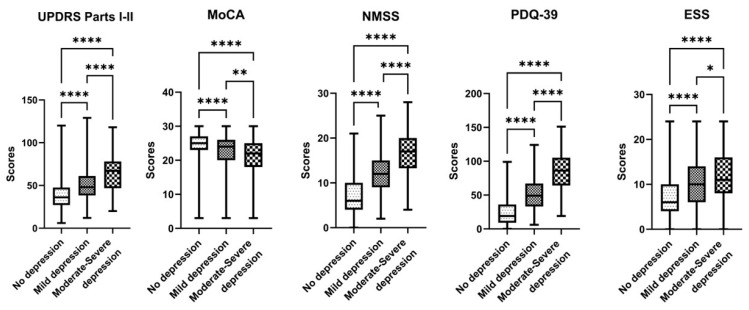
Boxplots for questionnaire scores and pairwise comparisons among the three Geriatric Depression Scale (GDS) groups. The number of asterisks reflects the number of decimal zeroes of the *p*-value, when *p* is statistically significant (*p* < 0.05), * *p* < 0.01, ** *p* < 0.001, **** *p* < 00001. UPDRS = Unified Parkinson’s Disease Rating Scale; MoCA = Montreal Cognitive Assessment; NMSS = Non-motor Symptoms Scale; PDQ-39 = Parkinson’s Disease Questionnaire; and ESS = Epworth Sleepiness Scale.

The mean (range) scores for all questionnaires (UPDRS, NMSS, MoCA, ESS, PDQ-39, and H&Y) are also reported in Table 1, both in the whole cohort and broken down by GDS groups. Overall, there was a trend towards worsening questionnaire scores with the increased depression scores. The UPDRS total score (Parts I–III) increased from a mean of 39 in the “no depression” group to 69 in the “moderate–severe depression group”. Similarly, the NMSS, ESS, PDQ-39, and H&Y scores worsened. The NMSS worsened from a mean of 7 in the “no depression” group to 16 in the “moderate–severe depression group”; The ESS worsened from 7 to 12; the PDQ-39 from 22 to 83. On the other hand, the MoCA scores decreased, indicating a worse mental status, from a mean of 24 in the “no depression” group to 21 in the “moderate–severe depression group”. The H&Y worsened from 2.0 to 2.5 and 2.9. We also added another analysis focusing only on PDQ-39 questions related to well-being (questions 17–22), and it worsened from 3 (no depression) to 9 and 15 (respectively, mild and moderate–severe depression; the Kruskal–Wallis Test was statistically significant). All comparisons among the three GDS groups were statistically significant, and all *p*-values were <0.01. The detailed boxplots for every GDS group based on the questionnaires are in Figure 1.

### 3.2. Logistic Regression Model for Depression Diagnosis

A multiple logistic regression model was created using the GDS score as the outcome/independent variable (Y) and the UPDRS, NMSS, MoCA, ESS, and PDQ-39 scores as the dependent variables (X), according to the formula:

GDS score~Intercept + UPDRS Parts I–III score + MoCA score + NMSS score + ESS score.

The logistic regression model indicated that for every 1-point increase in the NMSS score, the risk of depression (GDS ≥ 14) increased by 16%. On the other hand, for every 1-point increase in the PDQ-39 score, the risk of depression (GDS ≥ 14) increased by 5%. Both were statistically significant (*p* < 0.001). On the other hand, this model did not reach statistical significance when trying to diagnose depression based on the UPDRS Parts I–III (*p* = 0.14), MoCA (*p* = 0.59), or ESS (*p* = 0.20) scores.

**Figure 2:**
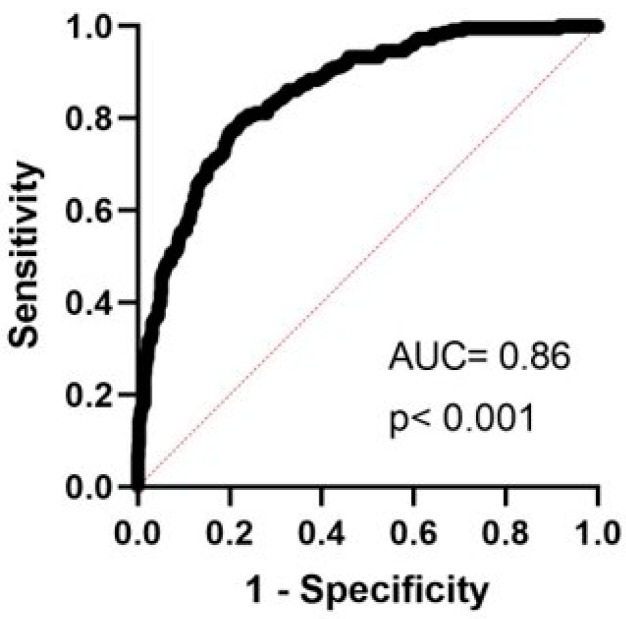
Area under the receiver operating curve (AUC) for the logistic regression model, using depression as the dichotomous independent variable (Geriatric Depression Scale, GDS ≥ 14 indicated depression, GDS < 14 indicates no depression), using the Unified Parkinson’s Disease Rating Scale Parts I–III, Montreal Cognitive Assessment, Non Motor Symptoms Scale, Parkinson’s Disease Quality of life-39, and Epworth Sleepiness Scale scores as dependent variables.

The model was promising overall in predicting the presence/absence of depression; the area under the receiver operating curve (AUROC, see Figure 2) was 0.86 (confidence interval 0.83–0.89, *p* < 0.0001, R-squared 0.35).

### 3.3. Medications Usage

As shown in Figure 1, the number of antidepressants taken at the initial evaluation was evaluated. There was an uptrend in the number of antidepressants (of any class) with greater depression. The percentage of PD patients using SSRIs increased, from “no depression” to “mild depression” and “moderate–severe depression”, from 12.7% to 25.0% and 33.3%, respectively. A similar trend was observed for SNRIs (from 4.3% to 8.8% and 18.4%), and “other antidepressants” (from 6.3% to 18.3% and 21.1%). 

Overall, the individuals with higher depression scores trended towards a higher proportion of antidepressant usage, with 46.5% of the “mild depression” and 59.6% of the “moderate–severe depression” patients taking an antidepressant, versus only about 21.3% taking antidepressants in the “no depression” group.

We also calculated the Levodopa Equivalent Daily Dose (LEDD) based on a conversion table already published [18]. Overall, we observed that the LEDD in our cohort was 440 (range 0–2800). Broken down by GDS groups, it was 396 (0–2800) in the “no depression” group, 496 (0–2246) in the “mild depression” group, and 570 (0–2000) in the “moderate–severe” depression group. The Kruskal–Wallis Test was statistically significant in this case (*p* < 0.001).

### 3.4. Comparing Questionnaire Scores in Patients Taking Antidepressants vs. Not Taking

Overall, the PD patients taking one or more antidepressants had significantly worse questionnaire scores, compared to those who were not taking antidepressants. The full results are reported in Table 2. The mean scores in the UPDRS, NMSS, MoCA, and PDQ-39 were significantly worse in those taking antidepressants: UPDRS Parts I–III (49 vs. 43), NMSS (12 vs. 9), PDQ-39 (50 vs. 34), H&Y (2.5 vs. 2.1), and MoCA (23 vs. 24). All *p*-values were <0.001.

## 4. Discussion

This database analysis found that depression as measured by the Geriatric Depression Scale (GDS) is common in PD, yet in a large number of patients it is not recognized, and, consequently, many patients with depression are not receiving treatment. Worsening depression positively correlated with a worsening of motor symptoms (according to the UPDRS), non-motor symptoms (according to the NMS questionnaire, ESS, and MoCA), and quality of life (according to the PDQ), based on their correspondent questionnaire scores. Therefore, as motor and other non-motor symptoms worsen, depression also appears to worsen. These findings suggest that, when clinical symptoms are worsening, it is important that depression is addressed.

The use of the GDS for evaluating depression in PD, among an array of alternative depression scales, is underpinned by a robust foundation that has already been demonstrated by a multitude of studies [19]. Unfortunately, there is often no time during clinic visits, especially in community settings, to administer a number of questionnaires. However, the assessment of motor symptoms is a routine part of a PD clinic assessment. Although the UPDRS is not always formally completed, a large portion is completed as part of a routine motor and non-motor assessment.

The UPDRS, introduced in 1987 [12], is a rating scale subdivided into four parts. The first segment focuses on mentation, the second focuses on ADLs and non-motor symptoms, the third on the classical motor features of PD, and the fourth on the motor complications of treatment. It is reasonable to suggest that worsening UPDRS scores, suggesting an increase in the motor and non-motor symptoms that result in greater disability, would increase depression.

We might also assume that depression might not exert a substantial influence on the UPDRS parts II and III due to their composition—these segments involve the objective or subjective assessment by patients and raters regarding the activities of daily function and motor examination. Although it is possible that the association between the UPDRS II and III scores and depression could be due to a worsening of PD symptoms, it is important to consider that worsening depression could also worsen motor symptoms due to psychomotor retardation and apathy and patients should not only be treated for the motor symptoms but also for depression.

Two studies have reported an analogous analysis to the present study [20,21], albeit employing a different approach. These studies included only those items from Part I of the UPDRS that were strongly consistent with depression screening, referred to as UPDRSd. They reported that “*…the GDS total score was largely accounted for by anxiety, apathy, and fatigue […] a smaller portion of variance accounted for by subjective cognitive functioning and depression (MDS-UPDRSd). Depression appeared to offer little predictive power…*” [20].

Steinstark et al. [21] reinforced the findings of this current study. They reported that the UPDRS Parts I–III scores displayed significantly worse scores in the minor depression group compared to the control/dysthimic patients, as well as in the major depression group compared to the control/dysthimic patients. However, they were not able to observe any significant difference between minor and major depression in PD patients. The difference between our study and theirs could be partially explained by the different criterion used to diagnose depression within PD. As reported in their limitations section, their methodology for diagnosing depression consisted in using the Clinical Depression Rating Scale and the Hamilton Depression Scale, which may have accounted for the higher frequency of major depression (30% in their cohort vs. 9.4% in our cohort). It could be possible that their major depression group of PD patients might have included some individuals that would have been categorized as mild depression in this present study. That could explain the nonsignificant difference between the UPDRS total scores in the minor and the major depression groups.

With regards to the strong association between deteriorating MoCA scores and increasing GDS scores, that was largely expected. This stems from the well-established understanding that depression and cognitive impairment, especially in the earliest stages, can be challenging to discern. Symptoms such as anhedonia, apathy, and irritability occur with both depression and cognitive impairment [22]. Depression in PD patients could arise due to the evident constraints on quality of life and motor symptoms. Yet, subjective or mild cognitive impairment in PD patients might be often misattributed to evolving dementia rather than depression [23]. Suicide rates among older adults are correlated with a lower MoCA score [23]. On a more physiopathologic and complex level, not only depression and the intrinsic neurodegeneration typical of PD can have an effect on cognitive functions but also the concurrent dopaminergic treatment. This theory is named the dopamine overdose hypothesis [24], and it postulates that individuals with unaffected (or relatively unaffected) ventral striatum and prefrontal areas might experience impaired cognitive functions due to an overdose of Dopamine in such structures, whereas individuals with a higher burden of neurodegeneration (e.g., a worse PD staging) will experience benefits from a cognitive standpoint by repleting the lacking Dopamine [25].

Likewise, the correlation between deteriorating PDQ-39 scores and elevated GDS scores was expected. The bidirectional correlation between declining quality of life—stemming from motor and non-motor symptoms, as well as stress on caregivers—and depression has already been documented in PD [26]. It is also known that non-motor symptoms exert a substantial impact on quality of life [26]. These current results indicated that a 1-point increase in the PDQ-39 and NMSS scores was associated with a 5% and 16% higher depression risk, respectively.

In addition, the robust correlation identified in this study between non-motor symptoms (expressed by the NMSS) and daytime sleepiness (expressed by the ESS) and depression should not be interpreted as a mere linear relationship, where an increase in symptoms equated to a greater depression.

The underlying etiology of depression in individuals with PD, while not fully elucidated, presents evidence hinting at the role of synuclein deposits propagation. It has been demonstrated that non-motor symptoms often predate the appearance of motor symptoms [27]. In fact, early non-motor symptoms may emerge decades prior to motor symptoms [27,28,29]. These early symptoms have been historically explained with the Braak’s staging of synuclein deposition [30]. According to that theory, the initial misfolded synuclein buildup might commence in peripheral structures involved in non-motor symptoms such as the olfactory system (hence the anosmia), the dorsal motor nucleus of the vagus nerve (hence the sympathetic denervation), and the enteric nervous system (hence the constipation). As the synuclein deposition advances in a prion-like fashion, more central structures are involved by the pathological misfolded protein, and structures such as the pons might get involved in stage 2 (the pons has been linked to sleep disorders such as REM sleep behavior disorder [31]). The next steps, according to Braak, would be the involvement of the substantia nigra in the third stage, which is historically causative of the parkinsonian motor symptoms, followed by mesocortical involvement (stage 4). Dysfunction within the mesocortical connections could be particularly important for depression. It has been shown through a functional MRI (fMRI) that dysfunctional mesocorticolimbic dopaminergic neurotransmission in PD patients with depression is significantly impaired compared to non-depressed PD patients [32]. This aligns with the fact that certain neuropsychiatric symptoms, such as depression [33] and anxiety [27], respond to dopaminergic treatment. Moreover, dopamine is known to be the neurotransmitter of gratification and rewarding; in this sense, the depletion of dopamine not only in the midbrain but also in the mesocorticolimbic structures might be causative of anhedonia and depression in PD.

Lastly, it has been suggested that performing “emotional training” by performing pleasant activities such as active theater participation might counteract such phenomena and, in turn, improve mood and well-being [5]. In this scenario, the amount of dysfunction generated by the synuclein deposition within such pathways involved in the control of mood might be indicative of a worse phenotype, when compared to those individuals who do not have depression. This might explain why individuals who are taking antidepressants exhibit worse questionnaire scores (with the only exception for ESS, as shown in Table 2).

There are limitations to this present study. These data were gathered by first-visit patients referred from the community, many of whom might have never consulted a movement disorder specialist or even a neurologist. In addition, a specific GDS cut-off for determining the presence and severity of depression was employed, rather than the more canonic use of the DSM-IV criteria. Another potential limitation is the fact that not all non-motor symptoms are present within the respective questionnaires; indeed, the impairment of inhibitory control (part of the dysexecutive symptoms [34]) has been reported as a potential indicator for the diagnosis and prognostication of PD [35] but is not tested within the NMSS. Lastly, we recognize the inherent limitations of patient-completed questionnaires including the subjectivity of the responses, the fact that some patients may underreport symptoms, and the impact of their cognitive status, cultural bias, and literacy level.

## 5. Conclusions

Depression in PD is a common non-motor symptom, potentially demonstrating a bidirectional relationship with most of the other symptoms encountered in PD, including motor and non-motor symptoms. Even though depression might naturally arise because of ongoing neurodegeneration, its association with synuclein on a microscopic pathological level should not be overlooked. Our ability to predict the presence of depression in PD based on NMSS and PDQ-39 scores should encourage clinicians to incorporate these questionnaires in their routine evaluations of PD patients. The worsening of motor functioning, as well as changes in non-motor symptoms and quality of life, should prompt clinicians to screen and offer treatment for depression.

## Figures and Tables

**Table 1:** Demographics and questionnaire scores of the full cohort of 1214 Parkinson’s disease patients, broken down by GDS group.

	No Depression (GDS 0–9)	Mild Depression (GDS 10–19)	Moderate-Severe Depression (GDS 20–30)	All Patients	*p*-Value
*N*	700	400	114	1214	
Gender (M/F)	449/251	247/153	63/51	759/455	
Age at onset—mean (min–max)	62.1 (27–88)	61.8 (33–87)	60.2 (34–80)	61.8 (30–88)	
Age at diagnosis—mean (min–max)	63.7 (30–88)	63.7 (35–91)	62.1 (36–82)	63.5 (34–91)	
UPDRS I—mean (min–max)	1.7 (0–11)	4.1 (0–12)	7.7 (0–15)	3.1 (0–15)	<0.001 *
UPDRS II—mean (min–max)	10 (0–38)	15 (1–47)	22 (1–46)	13 (0–47)	<0.001 *
UPDRS III—mean (min–max)	27 (3–78)	30 (5–73)	35 (9–62)	29 (3–78)	<0.001 *
UPDRS Parts I–III—mean (min–max)	39 (6–120)	50 (12–129)	65 (20–118)	45 (6–129)	<0.001 *
MoCA—mean (min–max)	24.4 (3–30)	22.9 (3–30)	20.7 (3–30)	23.6 (3–30)	<0.001 *
NMSS—mean (min–max)	7 (0–21)	12.1 (2–25)	16.3 (4–28)	9.7 (0–28)	<0.001 *
SSRIs	0 = 611	0 = 300	0 = 76	0 = 987	<0.001 *
	1 = 89 (12.7%)	1 = 100 (25.0%)	1 = 38 (33.3%)	1 = 227 (18.7%)	
SNRIs	0 = 670	0 = 365	0 = 93	0 = 1128	<0.001 *
	1 = 30 (4.3%)	1 = 35 (8.8%)	1 = 21 (18.4%)	1 = 86 (7.1%)	
Others	0 = 656	0 = 327	0 = 90	0 = 1073	<0.001 *
	1 = 40 (5.7%)	1 = 67 (16.8%)	1 = 22 (19.3%)	1 = 129 (10.6%)	
	2 = 4 (0.6%)	2 = 6 (1.5%)	2 = 2 (1.8%)	2 = 12 (1.0%)	
	1 + 2 = 44 (6.3%)	1 + 2 = 73 (18.3%)	1 + 2 = 24 (21.1%)	1 + 2 = 141 (11.6%)	
Any antidepressants	0 = 551	0 = 214	0 = 46	0 = 811	<0.001 *
	1 = 132 (19%)	1 = 161 (40%)	1 = 52 (46%)	1 = 345 (28%)	
	2 = 16 (2%)	2 = 22 (6%)	2 = 15 (13%)	2 = 53 (4%)	
	3 = 1 (0%)	3 = 3 (3%)	3 = 1 (1%)	3 = 5 (0%)	
	1 + 2 + 3 = 149 (21.3%)	1 + 2 + 3 = 186 (46.5%)	1 + 2 + 3 = 68 (59.6%)	1 + 2 + 3 = 403 (33.2%)	
PDQ-39—mean (min–max)	22.4 (0–99)	50.7 (6–124)	83.3 (19–151)	39.2 (0–151)	<0.001 *
ESS—mean (min–max)	7.2 (0–24)	10 (0–24)	11.9 (0–24)	8.6 (0–24)	<0.001 *
H&Y—mean (min–max)	2.0 (1–5)	2.5 (1–5)	2.9 (1–5)	2.3 (1–5)	<0.001 *
LEDD—mean (min–max)	396 (0–2800)	496 (0–2246)	570 (0–2000)	440 (0–2800)	<0.001 *

* statistically significant (*p* < 0.05). GDS = Geriatric Depression Scale; UPDRS = Unified Parkinson’s Disease Rating Scale; MoCA = Montreal Cognitive Assessment; NMSS = Non-motor Symptoms Scale; PDQ-39 = Parkinson’s Disease Questionnaire; ESS = Epworth Sleepiness Scale; H&Y = Hoehn and Yahr Scale; SSRI = Serotonin Selective Reuptake Inhibitor; SNRI= Serotonin and Norepinephrine Reuptake Inhibitor; and LEDD = Levodopa equivalent daily dose.

**Table 2:** Demographics and questionnaire scores of the full cohort of 1214 Parkinson’s disease patients, broken down by those using antidepressants vs. those not using.

	No Antidepressants (Min–Max)	Use Antidepressants (Min–Max)	*p*-Value
*N*	811	403	
Age at onset—mean (min–max)	62 (30; 80)	64 (34; 89)	-
Age at diagnosis—mean (min–max)	61 (34; 87)	63 (36; 91)	-
UPDRS I—mean (min–max)	2.5 (0; 14)	4.3 (0; 15)	<0.001 *
UPDRS II—mean (min–max)	12.0 (0; 43)	15.1 (0; 47)	<0.001 *
UPDRS III—mean (min–max)	28.0 (0; 78)	29.9 (0; 73)	0.002 *
UPDRS I–III—mean (min–max)	42.6 (8; 120)	49.4 (6; 129)	<0.001 *
MoCA—mean (min–max)	24 (3; 30)	22.8 (3; 30)	<0.001 *
NMSs—mean (min–max)	8.7 (0; 25)	11.8 (1; 28)	<0.001 *
PDQ—mean (min–max)	34.0 (0; 129)	49.8 (1; 151)	<0.001 *
ESS—mean (min–max)	8.4 (0; 24)	8.9 (0; 24)	0.10
H&Y—mean (min–max)	2.1 (1; 5)	2.5 (1; 5)	<0.001 *
LEDD (mg)—mean (min–max)	417 (0–2100)	487 (0–2800)	0.003 *

* statistically significant (*p* < 0.05). GDS = Geriatric Depression Scale; UPDRS = Unified Parkinson’s Disease Rating Scale; MoCA = Montreal Cognitive Assessment; NMSS = Non-motor Symptoms Scale; PDQ-39 = Parkinson’s Disease Questionnaire; ESS = Epworth Sleepiness Scale; H&Y = Hoehn and Yahr Scale; and LEDD = Levodopa equivalent daily dose.

## Data Availability

Dataset available on request from the authors.
